# Third-Space Entrapment of Methotrexate in Anthracycline-Induced Cardiomyopathy: The First Complete Case Trajectory From Toxicity to Rescue Failure

**DOI:** 10.7759/cureus.113550

**Published:** 2026-07-28

**Authors:** Sanchit Mehta, Syed Shahrukh Rizvi, Salimah Mohamed, Carlos Galvez

**Affiliations:** 1 General Internal Medicine, B.J. Medical College, Ahmedabad, IND; 2 Medicine, University of Illinois Chicago, Chicago, USA; 3 Medicine, Hematology, Oncology, University of Illinois Chicago, Chicago, USA

**Keywords:** anthracycline-induced cardiomyopathy, diffuse large b-cell lymphoma, doxorubicin, methotrexate toxicity, third-space entrapment

## Abstract

Methotrexate (MTX) is vital for treating multiple malignancies, including central nervous system (CNS) hematologic malignancies, but toxicity is a major limitation. Anthracyclines, cornerstones of lymphoma therapy, can cause cardiomyopathy, which may alter drug kinetics. This case highlights catastrophic MTX toxicity precipitated by prior anthracycline-induced cardiomyopathy.

A 64-year-old with diffuse large B-cell lymphoma (DLBCL) and anthracycline-induced cardiotoxicity was admitted for CNS-directed MTX therapy. Despite standard prophylaxis and prior tolerance, the patient developed anuria, hypotension, and cardiogenic shock following infusion. Serum MTX peaked at >600 μmol/L. Bedside imaging revealed a reduced ejection fraction and a large pleural effusion. Despite receiving glucarpidase and high-flux dialysis, MTX levels remained persistently elevated. Thoracentesis confirmed third-space sequestration with pleural MTX levels of 89 μmol/L. Clearance required 35 days of continuous dialysis and leucovorin rescue before levels fell below 0.1 μmol/L. The patient suffered cardiogenic shock and encephalopathy before transitioning to comfort care. This case illustrates how anthracycline-induced cardiomyopathy potentiates MTX toxicity through three mechanisms: decreased systemic circulation, cardiorenal syndrome-mediated reduced excretion, and increased third-space sequestration due to venous stasis. These factors trigger a deleterious cycle of multi-organ failure. This trajectory underscores the role of cardiomyopathy-induced effusions as reservoirs for third-space entrapment, which creates a pharmacological barrier leading to definitive rescue failure despite aggressive systemic detoxification and high-flux hemodialysis.

Clinicians must recognize cardiomyopathy as a critical risk factor for MTX sequestration and toxicity, regardless of whether prior cycles were tolerated. Early recognition of third-spacing and multidisciplinary management are essential to improve outcomes in high-risk cardio-oncology patients.

## Introduction

The foundation of induction therapy for primary central nervous system lymphoma (PCNSL) is high-dose methotrexate (HD-MTX), with different treatment regimens employing a variety of dosages and chemotherapy combinations [1]. The kidneys mainly remove methotrexate (MTX) through active tubular secretion and glomerular filtration. In acidic urine, MTX and its metabolites can precipitate to form crystals, increasing the risk of nephrotoxicity. To prevent this, urine alkalinization with intravenous sodium bicarbonate and aggressive hydration are standard supportive measures during high-dose MTX therapy. Maintaining strict urinary alkalinization enhances MTX solubility and clearance. Although high and frequent doses are required for central nervous system (CNS) penetration, doses greater than 500 mg/m² are linked to toxicities such as myelosuppression, mucositis, hepatotoxicity, pulmonary toxicity, nephrotoxicity, and an elevated risk of infection [2]. Supportive organ care, folinic acid (leucovorin) rescue, and improved drug clearance are all part of managing MTX toxicity. Patients with renal dysfunction and delayed MTX clearance ought to receive glucarpidase, especially if their serum MTX levels remain above standard toxic thresholds [3].

High-dose methotrexate can accumulate in extravascular fluid collections referred to as third spaces, such as pleural effusions, ascites, or periprosthetic seromas, which act as reservoirs leading to delayed drug clearance and elevated systemic toxicity risk. A retrospective analysis of osteosarcoma patients receiving HD-MTX showed MTX concentrations in periprosthetic seroma fluid were significantly higher than corresponding serum concentrations at 24 hours, reaching highly toxic levels [4]. Other studies confirm third space fluid collections correlate with delayed MTX elimination and increased toxicity. Clinicians should consider draining large effusions or seromas before MTX administration to avoid the third-space effect, although the risk of infection and feasibility must be weighed carefully [5].

Doxorubicin, a widely used anthracycline in hematologic and solid tumors, is limited by its dose-dependent cardiotoxicity, which can progress to symptomatic congestive heart failure (CHF) characterized by diastolic dysfunction, ventricular dilatation, and systolic impairment [6]. This cardiomyopathy can further induce renal impairment, hepatic congestion, and third spacing with pleural effusions and peripheral edema, all factors that may impair MTX clearance and contribute to toxicity. A detailed translational study demonstrated risk for heart failure increases significantly at cumulative doxorubicin-equivalent doses of 320 to 450 mg/m², with subclinical diastolic dysfunction occurring at lower doses around 120 to 190 mg/m² [7]. Clinical case reports highlight patients developing severe cardiogenic shock and multisystem congestion, including multiple body cavity effusions following cumulative doxorubicin doses of 450 mg/m²-supporting the link between anthracycline cardiotoxicity and third space formation [8]. All of this may hinder MTX clearance.

Diffuse large B-cell lymphoma (DLBCL), the most common non-Hodgkin lymphoma, is typically treated with immunochemotherapy regimens such as rituximab, cyclophosphamide, doxorubicin, vincristine, and prednisone (R-CHOP), which includes anthracycline therapy [9,10]. CNS relapse is treated with HD-MTX-based therapies often including rituximab and temozolomide for improved CNS penetration [11]. Comorbidities such as anthracycline-induced cardiomyopathy and third spacing complicate methotrexate pharmacokinetics and increase toxicity risk due to altered drug clearance. Although these interactions remain underexplored, treatment planning must account for third spacing and cardiac dysfunction. Our novel case report presents a patient experiencing severe methotrexate toxicity likely precipitated by pleural fluid third spacing secondary to anthracycline-induced cardiomyopathy and compromised renal function.

## Case presentation

Initial presentation and malignancy diagnosis

A 64-year-old male presented with fatigue, decreased appetite, weight loss, and an enlarged axillary lymph node. He was diagnosed with DLBCL. His significant comorbidities included heart failure with reduced ejection fraction (HFrEF), type 2 diabetes mellitus, hypertension, and diabetic neuropathy.

Initial DLBCL treatment

The patient received six cycles of polatuzumab vedotin, rituximab, cyclophosphamide, doxorubicin, and prednisone (pola-R-CHP) chemotherapy. He achieved complete remission approximately 18 months before the current admission. Following a brief remission of several months, the patient presented with symptoms concerning for CNS relapse; notably, anthracycline-induced cardiotoxicity manifested shortly after the completion of his frontline therapy.

Cardiac decompensation related to anthracycline exposure (two months before admission)

Two months before current admission, concurrent with evaluation for CNS relapse, cardiac evaluation revealed a severely reduced ejection fraction, representing a significant decline from a prior evaluation that demonstrated a preserved ejection fraction. This acute deterioration was attributed to doxorubicin-induced cardiotoxicity from prior pola-R-CHP treatment. The patient developed increased oxygen requirements, elevated lactate, and clinical signs concerning for cardiogenic shock. He was treated with intravenous furosemide, which yielded symptomatic improvement. A concurrent bicarbonate drip for urinary alkalinization was discontinued due to suspected exacerbation of fluid overload. Guideline-directed medical therapy (GDMT) for HFrEF was initiated with metoprolol succinate and losartan.

CNS relapse and MTX therapy initiation (two months before admission)

Two months before the current admission, the patient experienced CNS relapse with dural-based and leptomeningeal disease. Cerebrospinal fluid (CSF) analysis confirmed malignant lymphoid cells. The patient was initiated on the MTX protocol (high-dose methotrexate, temozolomide, rituximab) for CNS-directed chemotherapy.

Early MTX cycles (cycles 1 to 4)

The patient was admitted for cycles 1 to 4 at approximately 14-day intervals over the preceding two months. During cycle 3 of MTX therapy, the patient had transiently elevated methotrexate levels, which normalized to safe thresholds the following day without acute complications. Cycle 4 was administered 15 days prior to the current admission, without incident. GDMT was held during cycle 4 due to recurrent asymptomatic hypotension. Cardiac evaluation demonstrated a persistently reduced ejection fraction.

Severe MTX toxicity

Admission and Baseline Assessment (Day 0)

The patient was admitted for cycle 5, day 0, of HD-MTX. On examination, vital signs were stable and within normal limits on room air. Physical examination revealed an alert, thin gentleman in no acute distress, without jugular venous distension, edema, or respiratory symptoms. He remained alert and oriented to person, place, and time.

Baseline laboratory assessment prior to MTX infusion demonstrated a normal serum creatinine, though estimated glomerular filtration rate and creatinine clearance indicated mild preexisting renal impairment (stage 2 chronic kidney disease). These values represented borderline renal function that warranted cautious administration of high-dose methotrexate, which is primarily cleared renally. Complete blood count revealed mild anemia with normal white blood cell and platelet counts. The metabolic panel was largely unremarkable aside from mildly elevated blood urea nitrogen. Hepatic function testing showed elevated transaminases with normal bilirubin and intact synthetic function. Coagulation studies were unremarkable. Additional baseline abnormalities included markedly elevated lactate dehydrogenase, hyperuricemia, and an elevated anion gap. Despite baseline renal impairment, the patient was deemed eligible to proceed with cycle 5 HD-MTX infusion based on metabolic stability and acceptable renal function thresholds. MTX infusion was administered without incident on day 0.

Rapid Deterioration (Day 1)

**Figure 1:**
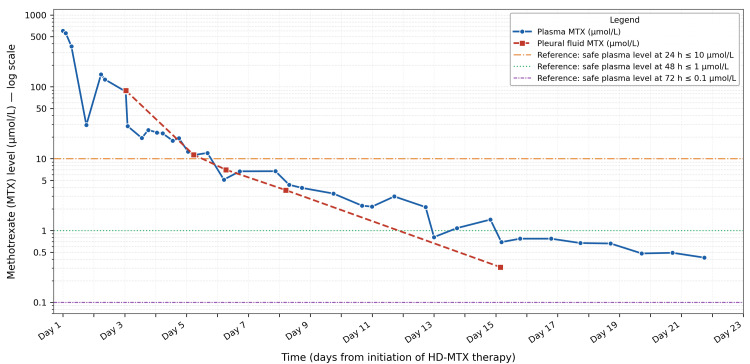
Plasma and Pleural Fluid Methotrexate Levels Over Time following High-Dose Methotrexate (HD-MTX) Therapy The graph depicts plasma (solid blue line, circle markers) and pleural fluid (dashed red line, square markers) methotrexate (MTX) levels measured serially over 22 days following high-dose methotrexate (HD-MTX) therapy. The Y-axis represents MTX concentration (µmol/L) on a logarithmic scale; the X-axis represents time in days from initiation of HD-MTX therapy. Reference thresholds for safe plasma methotrexate (MTX) levels are indicated as horizontal lines: ≤ 10 µmol/L at 24 hours (orange), ≤ 1 µmol/L at 48 hours (green), and ≤ 0.1 µmol/L at 72 hours (purple), as per standard HD-MTX toxicity monitoring guidelines. Figure generated using Microsoft Excel.

On the following day, his serum MTX level was noted to be critically elevated, with a repeat level later that day remaining severely high (Figure 1). His mental status changed dramatically to somnolent and confused. He became profoundly hypotensive with mean arterial pressure in the 50s mmHg, requiring dobutamine infusion. Cardiac monitoring revealed bigeminy and a right bundle branch block. He developed clinical evidence of bilateral pleural effusions. He developed acute kidney failure with anuria. The patient exhibited elevated lactate levels and signs of cardiogenic shock. He was transferred to the cardiology intensive care unit and started on urgent hemodialysis. Leucovorin rescue was initiated at the standard dose of 20-25 mg every six hours starting 24 hours after MTX infusion per MTX protocol. However, given the markedly elevated serum MTX level, which exceeded the standard 24-hour toxic threshold by 60-fold, leucovorin dosing was escalated to 40 mg every four hours. Additionally, Glucarpidase was ordered immediately. However, due to unavailability of the drug, administration was delayed until the following day. Glucarpidase was administered on day two, resulting in a significant decrease in methotrexate levels (Figure 1). However, the level rebounded the next day. Between days three and five, the patient underwent bedside thoracenteses, which correlated with progressive declines in methotrexate levels. Pleural fluid levels of methotrexate were measured and found to be severely increased (Figure 1). A specialized TheraNova High Flux Filter (Baxter International Inc., Deerfield, USA) was utilized by nephrology to assist in the clearance of methotrexate, with limited additional benefit.

Despite aggressive multidisciplinary interventions, the patient's clinical course remained complicated. During days 13-18, methotrexate fluctuated at low but persistently detectable levels with recurrent cardiogenic shock episodes. Between days 18-20, arrhythmia management escalated from intravenous amiodarone to milrinone due to dobutamine-induced tachyphylaxis. By days 20-52, the patient experienced intermittent altered mental status with recurrent shock episodes requiring ongoing vasopressor support. On day 53, midodrine dosing was increased to manage refractory hypotension. By day 54, worsening hypoxemia necessitated intubation due to increased supplemental oxygen requirements and persistent altered mental status. Between days 54-71, the patient developed severe hypoxic respiratory failure clinically consistent with acute respiratory distress syndrome. Despite maximal supportive care, the patient developed refractory hypotension requiring escalating pressors. On day 71 (hospital day 71) the patient was transitioned to comfort-focused care, and palliative extubation was performed (Figure 1).

## Discussion

This report appears to be the first to comprehensively characterize the full clinical trajectory and mechanistic underpinnings of catastrophic methotrexate toxicity due to anthracycline-induced cardiomyopathy, illustrating every major step from initial insult through rescue therapy failures and eventual outcomes. We describe a 64-year-old male with CNS DLBCL on MTX protocol who developed severe methotrexate toxicity during cycle 5 with acute kidney injury, altered mental status, and cardiogenic shock. He had recently developed severe doxorubicin-induced cardiomyopathy with a profoundly reduced ejection fraction, which appears to have altered MTX pharmacokinetics despite tolerating the same dose in four prior cycles.

Mechanism: third-spacing and delayed clearance

We hypothesize that anthracycline-induced cardiomyopathy led to profound third-spacing, creating pleural effusions and edema that served as methotrexate reservoirs, preventing systemic clearance even after glucarpidase treatment and hemodialysis. This is supported by: (1) significant MTX rebound after glucarpidase administration, suggesting reequilibration from pleural fluid; (2) severely elevated pleural MTX concentrations on thoracentesis; and (3) direct temporal correlation between pleural drainage and progressive serum MTX decline over consecutive days of drainage. Previous studies confirm that pleural effusions act as massive reservoirs for methotrexate, holding significantly higher concentrations of the drug than the serum at 24 hours. Consequently, the presence of these effusions more than doubles the drug's half-life [12].

Why cycle 5 was different

Rapid cardiac deterioration (from a preserved baseline to a severely reduced ejection fraction within eight months) combined with mildly reduced baseline creatinine clearance and elevated baseline lactate created a high-risk environment for MTX sequestration that was absent in earlier cycles. Additionally, cumulative MTX exposure from prior cycles may have caused subclinical tubular injury, reducing renal reserve and increasing susceptibility to acute kidney injury during cycle 5.

Cognitive errors and missed opportunities

Anchoring bias-one of the most common cognitive errors in medicine-occurs when clinicians rely too heavily on initial information, such as past successful cycles or previous lab findings, and fail to adequately adjust their thinking in response to new risk factors or clinical deterioration [13]. In this case, the team may have anchored on the tolerance of the prior four cycles of methotrexate, potentially overlooking the significance of newly developed severe cardiomyopathy and renal impairment. Premature closure compounded the error by accepting reassuring initial findings, rendering the team less likely to recognize early red flags in laboratory values and symptoms. Process errors such as search satisficing-where clinicians cease further investigation once a plausible solution is found-can lead to premature decisions and delay critical interventions like dialysis or drainage. Feedback sanction refers to situations where lab result delays or slow feedback loops hinder timely toxicity recognition and treatment adjustment, ultimately worsening outcomes. Such reasoning pitfalls are well described in medical literature and highlight the need for continuous reassessment and improved systems for rapid feedback and multidisciplinary action. Studies show that such biases are common, particularly under stress or time pressure, and can directly cause missed opportunities for risk reassessment, consultant involvement, and changes in management [14]. Systematically involving multidisciplinary teams early can mitigate anchoring bias and improve patient safety, especially for high-risk oncology patients with evolving comorbidities. Lack of multidisciplinary involvement here meant cardiology was consulted reactively rather than proactively before cycle 5. Key missed opportunities: 1. cardiology consultation before MTX administration; 2. baseline clinical assessment for third-spaced fluids; 3. glucarpidase availability planning; 4. recognition of mildly reduced creatinine clearance as a significant risk factor.

System failures and root cause analysis

Glucarpidase was ordered immediately, but administration was delayed ~24 hours due to institutional supply limitation. In several reports, timely access to glucarpidase was challenging due to institutional stock issues and financial constraints, resulting in the use of reduced or partial doses-sometimes less effective-and delayed toxicity reversal in patients at risk. Such cases highlight the real-world barriers hospitals face in rapidly procuring expensive rescue medicines during acute emergencies [15]. Most critically, our case demonstrates that while institutions appropriately restrict anthracyclines in cardiotoxic patients, similar vigilance is not uniformly applied to other drugs with high tissue distribution (MTX), even though altered pharmacokinetics may increase toxicity risk substantially. Furthermore, comprehensive consensus guidelines by Ramsey et al. emphasize that glucarpidase is most effective when administered within 48 to 60 hours from the start of the methotrexate infusion [16]. Our patient's clinical trajectory aligns with their findings that when administration is delayed beyond this critical window, as occurred in our case due to logistical procurement barriers, life-threatening systemic toxicities become irreversible despite eventual enzymatic cleavage of the drug in the vascular space.

Leucovorin and glucarpidase rescue failure

In this patient, both glucarpidase and leucovorin provided only transient reductions in plasma methotrexate due to ongoing third-space redistribution from pleural effusions and severe renal failure. The efficacy of glucarpidase diminishes when administration is delayed, or drug sequestration persists, and leucovorin cannot adequately neutralize high-dose MTX outside the vascular compartment or in the context of compromised renal function. Regarding extracorporeal clearance, prior systematic evaluations of high-flux hemodialysis for methotrexate toxicity demonstrate that while plasma levels may drop acutely during the dialysis session, significant and rapid rebound inevitably occurs due to redistribution from deep tissue compartments [17]. Our patient's experience with the TheraNova High Flux Filter mirrors these literature findings, reinforcing that extracorporeal removal cannot independently overcome the pharmacokinetics of massive third-space sequestration, especially when compounded by cardiogenic shock.

Literature comparison

While delayed methotrexate clearance due to third-space fluid compartments has been described in other oncologic contexts-such as osteosarcoma with periprosthetic seromas and multisystem effusions in advanced anthracycline cardiomyopathy-this report is novel in fully characterizing the mechanistic progression, clinical trajectory, and therapeutic challenges of catastrophic methotrexate toxicity in the setting of severe anthracycline-induced cardiac dysfunction [4]. Additionally, comprehensive guidelines establish strict qualitative safe clearance thresholds for high-dose methotrexate at 48 and 72 hours (as detailed in Figure 1). While previous retrospective cohorts have established that even mild preexisting renal impairment significantly prolongs initial methotrexate excretion [18], our case adds a critical dimension to this literature. It illustrates how the compounding mechanical effect of massive third-spacing completely overrides standard pharmacologic rescue protocols, transforming a known renal risk factor into a fatal systemic cascade that exponentially exceeded these safe prognostic limits.

Limitations

A single-center case report limits generalizability. Competing etiologies are not excluded, though third-space sequestration is most parsimonious given the thoracentesis-MTX correlation.

## Conclusions

Catastrophic methotrexate toxicity in cardio-oncology patients is primarily driven by third-spacing and delayed drug clearance. Patients with prior anthracycline-induced cardiomyopathy are at especially high risk, as altered cardiac function profoundly impacts methotrexate pharmacokinetics-even with doses previously tolerated. Clinicians must rigorously reassess risk before each chemotherapy cycle, preemptively evaluating for sites of potential third-spacing and recognizing transient methotrexate elevations as a warning sign to avoid supportive care measures that may worsen fluid overload in severe cardiomyopathy. Early thoracentesis, timely glucarpidase administration, and mandatory multidisciplinary involvement are essential to prevent and manage toxicity effectively. Future research should focus on identifying high-risk patients and optimizing intervention timing to improve outcomes.
